# *Citrus aurantium* Naringenin Prevents Osteosarcoma Progression and Recurrence in the Patients Who Underwent Osteosarcoma Surgery by Improving Antioxidant Capability

**DOI:** 10.1155/2018/8713263

**Published:** 2018-02-07

**Authors:** Lirong Zhang, Xiaohua Xu, Tiechao Jiang, Kunzhe Wu, Chuanbo Ding, Zhen Liu, Xuanhe Zhang, Tianhua Yu, Changlong Song

**Affiliations:** ^1^Department of Pathology, China-Japan Union Hospital of Jilin University, Changchun 130033, China; ^2^Department of Nephrology, China-Japan Union Hospital of Jilin University, Changchun 130033, China; ^3^Cardiology Department, China-Japan Union Hospital of Jilin University, Changchun 130033, China; ^4^Department of Gastrointestinal Surgery, The Fourth Affiliated Hospital of China Medical University, Shenyang 110032, China; ^5^Teaching Office, College of Chinese Medicinal Materials, Jilin Agricultural University, Changchun 130118, China; ^6^Pediatrics, Liuhe District Hospital of Nanjing, Nanjing 211500, China; ^7^Shihezi University, Shihezi 832003, China; ^8^Blood Transfusion Department, China-Japan Union Hospital of Jilin University, Changchun 130033, China; ^9^Breast Surgery Department, China-Japan Union Hospital of Jilin University, Changchun 130033, China

## Abstract

*Citrus aurantium* is rich in flavonoids, which may prevent osteosarcoma progression, but its related molecular mechanism remains unclear. Flavonoids were extracted from *C. aurantium* and purified by reparative HPLC. Each fraction was identified by using electrospray ionisation mass spectrometry (ESI-MS). Three main components (naringin, naringenin, and hesperetin) were isolated from *C. aurantium*. Naringenin inhibited the growth of MG-63 cells, whereas naringin and hesperetin had no inhibitory function on cell growth. ROS production was increased in naringin- and hesperetin-treated groups after one day of culture while the level was always lowest in the naringenin-treated group after three days of culture. 95 osteosarcoma patients who underwent surgery were assigned into two groups: naringenin group (NG, received 20 mg naringenin daily, *n* = 47) and control group (CG, received 20 mg placebo daily, *n* = 48). After an average of two-year follow-up, osteosarcoma volumes were smaller in the NG group than in the CG group (*P* > 0.01). The rate of osteosarcoma recurrence was also lower in the NG group than in CG group. ROS levels were lower in the NG group than in the CG group. Thus, naringenin from *Citrus aurantium* inhibits osteosarcoma progression and local recurrence in the patients who underwent osteosarcoma surgery by improving antioxidant capability.

## 1. Introduction

Osteosarcoma is a malignant tumor affecting bone metaphysis and patient life quality. The present therapeutic approach of osteosarcoma is neoadjuvant chemotherapy following surgical resection of primary tumors. The estimated osteosarcoma morbidity is more than four cases for per million population worldwide [1]. With chemotherapy and surgery, the five-year survival rate of the patients without fracture is more than 50% [2]. Unfortunately, survival rates remain unchanged over the past decades because no new drug is available. Furthermore, some side effects are often caused by medical treatment [3, 4]. At present, some treatment modalities can be available for osteosarcoma. For instance, mifamurtide (produced by Takeda) is a drug against osteosarcoma and was approved in Europe in March 2009. The drug can be used for treating high-grade, nonmetastasizing, and resectable osteosarcoma after surgical removal [5]. The normal side effects of mifamurtide include fever, vomiting, fatigue and tachycardia, infections, anaemia, anorexia, headache, and constipation [6, 7]. Therefore, it is necessary to find new medicine from functional food with few side effects.

Citrus is a genus of flowering trees and shrubs in Rutaceae. *Citrus aurantium*, as the “Chinese bitter orange,” is native to Southeast Asia and has been spread worldwide. The species is widely used in traditional medicine as a medicinal herb in Asian countries. Due to its antitumor activities with few side effects, citrus juice is a therapeutic agent against meningioma cells [8]. Citrus fruit intake has been reported to control the risks of breast cancer [9] and stomach cancer [10]. The antiproliferative and apoptotic effects of flavonoids isolated from *C. aurantium* have been found in lung cancer cells [11] and gastric cancer cells [12]. Naringenin has been proven to inhibit the growth and induce autophagy of colon cancer cells by affecting isoprenylcysteine carboxyl methyltransferase/RAS signaling [13]. Further work has proved that naringenin is a potential chemotherapeutic agent to suppress the progression of prostate cancer cell lines via the phosphatidylinositol 3 kinase (PI3K)/Akt and mitogen-activated protein kinase signaling [14]. Naringenin as the main component of citrus fruit inhibits the expression of epidermal growth factor receptor-2 and promotes cancer death [15].

The consumption of orange juice has been reported to increase the plasma concentration of vitamin C, folate, carotenoid, and flavanone and hydrophilic and lipophilic phytochemical [16]. Many small molecule candidates have been found to target metastatic osteosarcoma-associated subpathways. Lansoprazole is supposed to treat metastatic osteosarcoma [17]. However, the effects of *C. aurantium* on osteosarcoma are seldom reported and an exact molecular mechanism is still needed to be elucidated.

*C. aurantium* is rich in flavonoids [18, 19], which have antioxidants [20, 21] and anti-inflammation properties [22]. Reactive oxygen species (ROS) are closely associated with oxidative stress, which is ubiquitous in a biological system. ROS are normally produced by chemical, photochemical, electronic activities, endogenous enzymatic reactions, and inflammation [23]. High-level ROS will increase oxidative stress, resulting in deleterious oxidation and chemical modification and cancer risk [24]. In contrast, a certain level of ROS production may be beneficial to control cancer progression by inducing apoptosis in cancer cells [25]. In the case, the flavonoids from *C. aurantium* may have inhibitory functions on osteosarcoma progression by affecting ROS levels. Therefore, we investigated the effects of flavonoids from *C. aurantium* on osteosarcoma and specific bioactive component was confirmed. Inhibitory functions of *C. aurantium* were further identified on osteosarcoma patients who underwent surgery by investing osteosarcoma volume, local recurrence, and antioxidant properties.

## 2. Materials and Methods

### 2.1. Flavonoids Extracts from *C. aurantium* Peel

Fresh *C. aurantium* fruit was purchased from the local supermarket and the peel was isolated from the fruit. Twenty-gram fruit peel was comminuted and then subjected to extraction with 200 mL 20% ethanol for 4 h. The mixture was filtrated with a membrane NMWL of 3 kDa (Millipore, Billerica, MA, USA). The filtrated liquid was dried and dissolved in 20 *μ*L methanol.

### 2.2. Preparative Reversed-Phase HPLC

One mL above aliquot was injected into a preparative HPLC (Gilson, Middleton, WI, USA). The column was eluted with 30% ethanol and linearly increased to 100% at a flow rate of one mL/min. The peaks were detected at 360 nm. According to the peak time, each individually identifiable peak was collected by using a detector. The collected fraction was dried and resolved in 20 *μ*L ethanol.

### 2.3. Mass Spectrometry

Each fraction was analyzed by Micromass ESI Mass Spectrometer (Waters). Source temperature was 100°C, and the desolation gas temperature was 350°C. The purity of nitrogen and argon was over 99.99%. Desolation gas flow was 650 L/h and cone gas flow was 50 L/h, respectively. The sampling cone was 25 V and the capillary voltage was 3 kV. Sodium format was used to calibrate mass spectrometer in both positive- and negative-ion modes. The mass spectrometer was set over the range *m*/*z* 0–600.

### 2.4. Cell Culture

Human osteosarcoma cell line MG-63 was purchased from the cell bank of Chinese Academy of Sciences (Shanghai, China) and cultured in DMEM at 37°C with 5% CO_2_. After three-day culture, cell concentrations were adjusted to 1 × 10^5^ cells/mL and 100 *μ*L cells were transferred to each cell of a 96-cell plate, treated with different fractions of crude extracts, and further cultured for three days under the same situation. Cell growth was recorded by using RTCA iCELLigence (ACEA Biosciences Inc., San Diego, CA, USA).

### 2.5. Participants

From May 4, 2011 to March 13, 2014, osteosarcoma patients who underwent limb salvage surgery and osteosarcoma removal were recruited at our hospital. This randomized prospective study was performed. All procedures were approved by the ethics committee of Jilin University (Changchun, China). The written informed consent was obtained from the patients of this study.

### 2.6. Inclusion Criteria

All patients were recruited according to the following inclusion criteria: (1) the patients had a histological diagnosis of osteosarcoma by biopsy, (2) the patients were treated with the combination of chemotherapy and surgery, and (3) the patients were from the same city, Changchun, China.

### 2.7. Exclusion Criteria

The patients underwent amputation surgery; the patients received radiotherapy; the patients died within three months after being recruited; and the patients were pregnant.

### 2.8. Patient Grouping

Twenty-five g of mannitol, 5 g PEG6000, 50 g naringenin, 35 g meglumine in povidone K30 ethanol solution (50% (*v*/*v*) were mixed well and wet granulated. The mixture was dried at a reduced pressure less than 60°C oven and compressed into tablets after adding 3.5 g sodium carboxymethyl starch and 1 g of magnesium stearate. Meanwhile, the placebo tablets were made by using the same method.

After the selection of inclusion criteria and exclusion criteria, a total of 95 osteosarcoma patients were selected in this study. Forty-seven patients received the tablets with 20 mg naringenin daily and assigned as an NG group. Forty-eight patients received the tablets with 20 mg placebo daily and assigned as a CG group. All demographic data were normalized between the two groups. Their preoperative clinical data and operative information were recorded. According to Broders' criteria, osteosarcoma can be divided into G1, G2, G3, and G4 grades [26]. Osteosarcoma volumes were measured by using magnetic resonance (MR) images and calculated via the formula: ([*π*/6] × length × width × depth). The evaluation of all patients was based on radiology findings. The whole follow-up period was two years, and osteosarcoma volume and local recurrence were investigated. Osteosarcoma samples at different stages were stained with hematoxylin and eosin stain (H&E) according to a previous report [27]. The therapy of osteosarcoma patients was performed according to a previous report with modification [28]. All osteosarcoma patients met the inclusion criteria and received surgery compliance with chemotherapy as Table 1 showed.

### 2.9. Diet Comparison between NG and CG

Considering the effects of patient diets on osteosarcoma progression, the overall dietary consumption of food and beverages consumed by all osteosarcoma patients was evaluated between NG and CG groups by using a food frequency questionnaire (FFQ). Nutrition experts administered the FFQ for interviewing osteosarcoma patients. The FFQ included the following items: (1) how often did you consume food (times daily, weekly, monthly, and yearly) during the past two years? (2) Amount of monthly intake of major fruit, including citrus, apple, pear, peach, banana, plum, grape, litchi, papaya, and watermelon; (3) amount of monthly intake of major vegetables, including carrot, lettuce, Chinese cabbage, cauliflower, celery, lotus root, potato, onion, garlic, bean and its products, pumpkin, cucumber, eggplants, peppers, tomato, turnip, radish, and mushroom; (4) amount of monthly intake of major nuts, including peanut and cashew; (5) amount of monthly intake of protein, including beef, lamb, pork, fish, eggs, and poultry; (6) amount of monthly intake of carbohydrate, including barley, rice, corn, oats, sugar, taro, potato, and wheat; (7) amount of monthly intake of animal fats and vegetable oils; and (8) amount of monthly intake of beverages. Daily consumption of these diets was calculated as g/day. Energy and nutrient intakes were assessed by using the food composition.

### 2.10. Cell-Based Bioassay of Meals

Arising from other components present as well as overall food matrix effects, the effects of the meals on naringenin function were measured. Breakfast, lunch, and dinner were randomly obtained from individual suppliers of osteosarcoma patients. Each meal was ground with an agate mortar and pestle on ice. The mixed meal was sterilized at 60°C for 60 min and added to MG-63 cells at 10 mg/mL. The effects of meals from NG and CG, with naringenin or placebo, on the growth of MG-63 cells were measured.

### 2.11. Measurement of the Plasma and Urine Levels of Naringenin

The plasma and urine levels of naringenin in both their NG and CG groups were compared according to previously reported methods [29, 30]. Briefly, 2 mL urine was obtained from each patient and centrifuged at 12,000*g* for 10 min. 0.25 mL supernatant was taken and mixed with 50 *μ*L of 0.5 M triethylamine acetate (pH 7.0), 5 *μ*L *β*-glucuronidase, and arylsulfatase, respectively. The mixture was incubated for 60 min at 37°C. The urine was extracted with 2.0 mL ethyl ether and 50 *μ*L formononetin (10 ppm in methanol) was used as an internal control. The extract was dried by using a freeze drier and dissolved in 0.25 mL methanol. Subsequently, 0.25 mL 0.2 M acetate buffer (pH 5) was added and 10 *μ*L mixture was injected into the HPLC system. Five mL was obtained from each patient and plasma was separated by blood centrifugation for 5 min at 1000*g*. One hundred *μ*L of 200 mM triethylamine buffer (pH 7.5) was added to 500 *μ*L serum or plasma and incubated with 45 *μ*L *β*-glucuronidase and 45 *μ*L of arylsulfatase for 15 h at 37°C. Twenty *μ*L formononetin (10 ppm in methanol) was used as an internal control. Targeted fractions were extracted by adding two mL of diethyl ether. The extracts were dried by using a freeze drier and dissolved in 100 *μ*L methanol and diluted with 100 *μ*L of 200 mM acetate buffer (pH 5.2). Ten *μ*L of the mixture was injected into HPLC system an HPLC (Gilson, Middleton, USA). Separation was performed on a Phenomenex Luna C8 column (250 × 30 mm, 5 *μ*m C8). The mobile phase was methanol/water/acetic acid (40 : 58 : 2, *v*/*v*/*v*). The flow rate was set at one mL/min at 46°C and the detector was set at 290 nm. Stock solutions of naringenin and internal standard 7-ethoxycoumarin were prepared at 500 *μ*g/mL, respectively. Stock solution of naringenin was diluted with methanol/water (1 : 1, *v*/*v*) to 0.5, 1, 2, 4, 8, and 16 *μ*g/mL. The standard was diluted with methanol/water to 20 *μ*g/mL. Urine and plasma were calculated according to the standard curve.

### 2.12. Biochemical Analysis

Superoxide dismutase (SOD) [31, 32], aspartate aminotransferase (AST) [33], alanine aminotransferase (ALT) [34], and glutathione (GSH) are associated with oxidative stress [35]. Here, serum levels of SOD, AST, ALT, and GSH were measured. Serum AST and ALT were measured by an automated clinical chemistry analyzer (Indianapolis, IA, USA). SOD activity was measured by a SOD Assay Kit (Cat. number 19160-1KT-F from Sigma, St. Louis, MO, USA). GSH was measured by a GSH Assay Kit (Cat. number CS0260-1KT from Sigma).

Lipid profiles are closely associated with osteosarcoma risks. Thus, the serum lipid patterns, including total triglycerides (TG), total cholesterol (TC), high-density lipoprotein cholesterol (HDL-C), and low-density lipoprotein cholesterol (LDL-C), were measured by an automatic biochemical analyzer (Beckman Coulter Inc., Brea, CA, USA). Malondialdehyde (MDA) level was measured by an MDA assay kit (Cat. number MAK085-1KT from Sigma).

### 2.13. Measurement of Inflammatory Cytokines

Osteosarcoma pathology is closely associated with the levels of interleukin-1 (IL-1) beta [36] and IL-6 [37]. Five mL blood samples were obtained from all participants. Thus, the levels of these cytokines were measured by using human ELISA kits for IL-1 beta (Cat. number KHC0011) and IL-6 (Cat. number KHC0061) from Thermo Fisher Scientific (Hong Kong) Limited.

### 2.14. Measurement of ROS

To measure serum ROS level, 5 mL blood was taken from each patient by using a vacuum tube. Serum was isolated following a centrifugation at 2000 rpm for 10 min. Serum ROS was measured by using dROMs test (Diacron International, Hong Kong) [38] on the F.R.E.E. analyzer (Diacron).

Aringin, naringenin, and hesperetin were added to MG-63 cells at 1 *μ*g/mL, respectively. MG-63 cells (1 × 10^5^ cells/mL) were transferred to a 24-well plate for one day. The assay was performed according to a previous report [39]. To measure SOD level in interval cells, the cells were treated with 2′,7′-dichlorodihydrofluorescein diacetate (DCFH-DA) for 1 h at 37°C under 5% CO2, and DCFH-DA reacts with ROS to produce 2′, 7′-dichlorofluorescein (DCF), an indicator of general oxidative stress. The fluorescence intensity was detected under a confocal fluorescence microscope and a flow cytometer at an excitation and emission wavelength of 488 nm and 535 nm. Data were analyzed by using FV10-ASW software (version 4.2; Olympus Corporation, Tokyo, Japan).

### 2.15. Statistical Analysis

Student's *t*-test was used to compare the significant difference in variable data between the two groups. Chi-square test was used to compare the significant difference in the numbers between the two groups. Statistical data were analyzed by using the SPSS 20.0 (SPSS Inc., Chicago, IL, USA). There are statistically significant differences if *P* < 0.05.

## 3. Results

### 3.1. Characterization of *C. aurantium* Extracts

**Figure 1:**
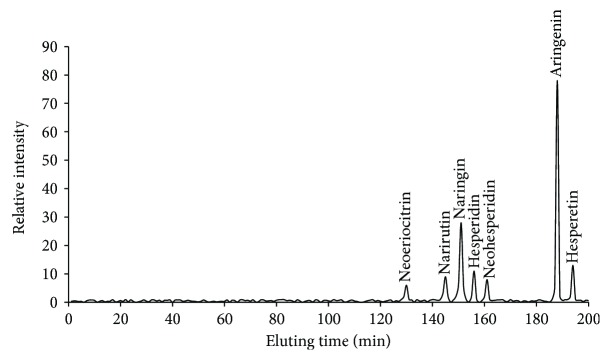
HPLC analysis of the main components of the extracts of *Citrus aurantium*. Peak label shows corresponding components.

**Figure 2:**
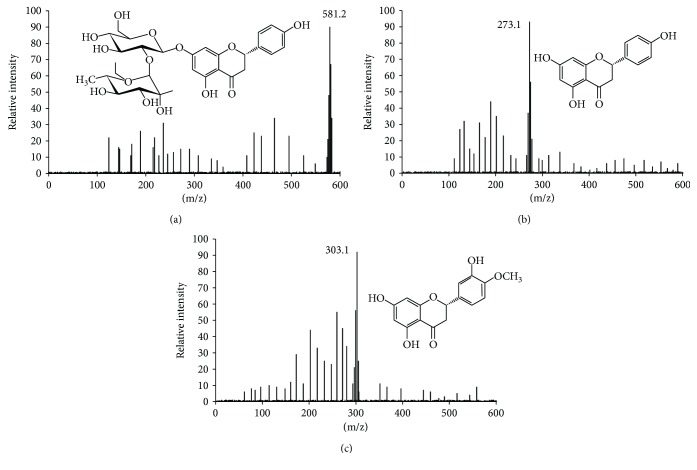
ESI MASS spectrometry analysis of bioactive fractions from the extracts of *Citrus aurantium* under the conditions that produced mass spectra with [M + H]^+^. (a) Mass spectra were visualized following the separation of naringin ([M + H]^+^ = 581 Da). (b) Mass spectra were visualized following the separation of naringenin ([M + H]^+^ = 273 Da). (c) Mass spectra were visualized following the separation of hesperetin ([M + H]^+^ = 303 Da).

Three main components (mg/100 g peel, naringin 8, naringenin 27, and hesperetin 3) were isolated from *C. aurantium* crude extracts (10 mL/100 g peel) after HPLC separation (Figure 1) and further confirmed by electrospray ionization (ESI) mass spectrometry under the conditions that produced mass spectra with [M + H]^+^. Figure 1 shows that the predicted masses for naringin (Figure 2(a)) and naringenin (Figure 2(b)) and hesperetin (Figure 2(c)) were 580, 272, and 302 Da, respectively. Naringin, a flavanone-7-O-glycoside (Figure 2(a)), existed in citrus fruit with the fruit's bitter taste. Naringenin, a flavonoid ((S)-2,3-dihydro-5,7-dihydroxy-2-(4-hydroxyphenyl)-4-benzopyrone, Figure 2(b)) in citrus, has been reported to have antioxidant, antiatherogenic, and anticancer properties [40]. Hesperetin, the 4′-methoxy derivative of eriodictyol (Figure 2(c)), is a naturally occurring flavanone-glycoside in citrus [41]. Hesperetin has been proven to have antiproliferative ability against colon tumorigenesis [42].

### 3.2. Naringenin Inhibits the Growth of MG-63 Cells

**Figure 3:**
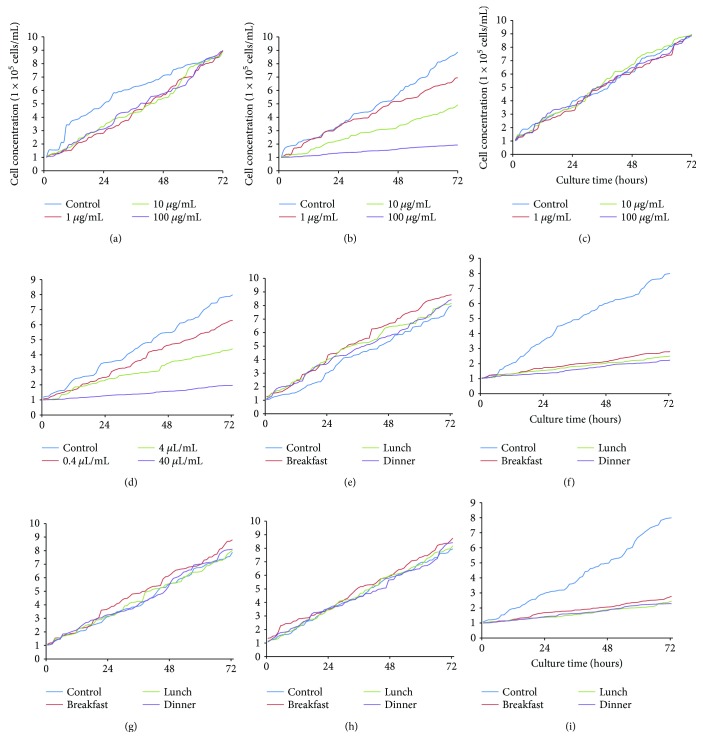
Real-time analysis for the effects of bioactive components of *Citrus aurantium* on the growth of MG-63 cells. (a) The effects of naringin on the growth of MG-63 cells. (b) The effects of naringenin on the growth of MG-63 cells. (c) The effects of hesperetin on the growth of MG-63 cells. (d) Crude extracts of *Citrus aurantium* on the growth of MG-63 cells. (e) The effects of meals of NG group on the growth of MG-63 cells. (f) The effects of meals of NG group and 100 *μ*g/mL naringenin on the growth of MG-63 cells. (g) The effects of meals of CG group on the growth of MG-63 cells. (h) The effects of meals of CG group and 100 *μ*g/mL placebo on the growth of MG-63 cells. (i) The effects of meals of CG group and 100 *μ*g/mL naringenin on the growth of MG-63 cells.

Figure 3(b) shows that naringenin inhibited the growth of MG-63 cells with the increase in its concentration. Comparatively, naringin and hesperetin (Figures 3(a) and 3(c)) did not affect the growth rate of MG-63 cells. On the other hand, crude extracts of *C. aurantium* inhibited the growth of MG-63 cells with the similar results as only naringenin (Figure 3(d)). These results suggested that it was naringenin rather than possible other components of crude extracts inhibiting the growth of osteosarcoma cells, and naringenin was therefore used in subsequent experiments. Arising from other components present as well as overall food matrix effects, the effects of daily diets with naringenin and placebo on the growth of MG-63 cells were also measured. The findings demonstrated that the meals of NG group did not affect the growth of MG-63 cells (Figure 3(e)). In contrast, the meals of NG group affected the growth of MG-63 cells when naringenin was added (Figure 3(f)). Similarly, the meals of CG group did not affect the growth of MG-63 cells (Figures 3(g) and 3(h)). The meals of CG group would not affect the growth of MG-63 cells until naringenin was added (Figure 3(i)).

### 3.3. Baseline Characters of Participants

A total of 95 patients had osteosarcomas and received surgeries. The average ages were 23.6 ± 12.7 and 24.3 ± 12.1 years in NG and CG groups, respectively. The most common sites of osteosarcoma occurred at the distal end of the femur. The average follow-up was 24 months (ranging from 6 to 80 months). All other parameters, including osteosarcoma grades and treatment types, were similar between NG and CG groups (Table 2, *P* > 0.05). Osteosarcoma resection margins are classified as follows: R0, no cancer cells can be seen microscopically at the resection margin; R1, cancer cells are presented microscopically at the resection margin; and R2, tumor tissues can be observed by the naked eyes at the resection margin. The statistical difference was insignificant for the resection status in the two treatment groups (R0/R1/R2, 6/33/8 in an NG group and 7/36/6 in a CG group, *P* = 0.80).

All the patients consumed food three times daily. The daily intake of food components was similar between NG and CG groups (*P* > 0.05, Table 3). Thus, the composition of meals would not cause the difference for osteosarcoma growth and recurrence.

**Figure 4:**
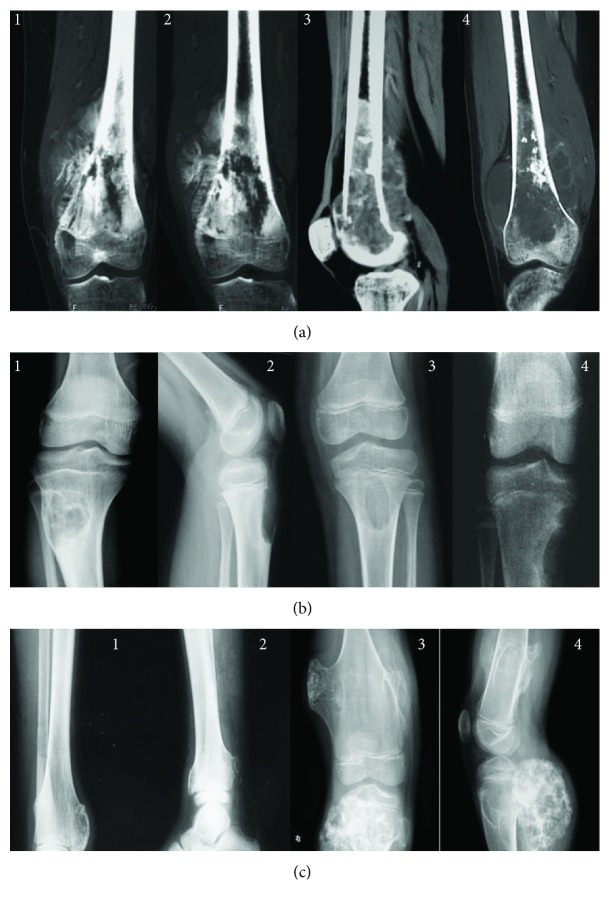
Radiological review of osteosarcoma at different stages. (a) Conventional/classic central osteosarcoma. (b) Chondromyxoid fibroma. (c) Small cell osteosarcoma. The numbers 1, 2, 3, and 4 stand for G1, G2, G3, and G4 osteosarcomas.

Figure 4 shows the radiological review of osteosarcoma at different stages. The size of conventional/classic central osteosarcoma (Figure 4(a)) and small cell osteosarcoma (Figure 4(c)) was increased with osteosarcoma progression. For chondromyxoid fibroma, bone intensity was reduced significantly with the osteosarcoma development (Figure 4(b)).

**Figure 5:**
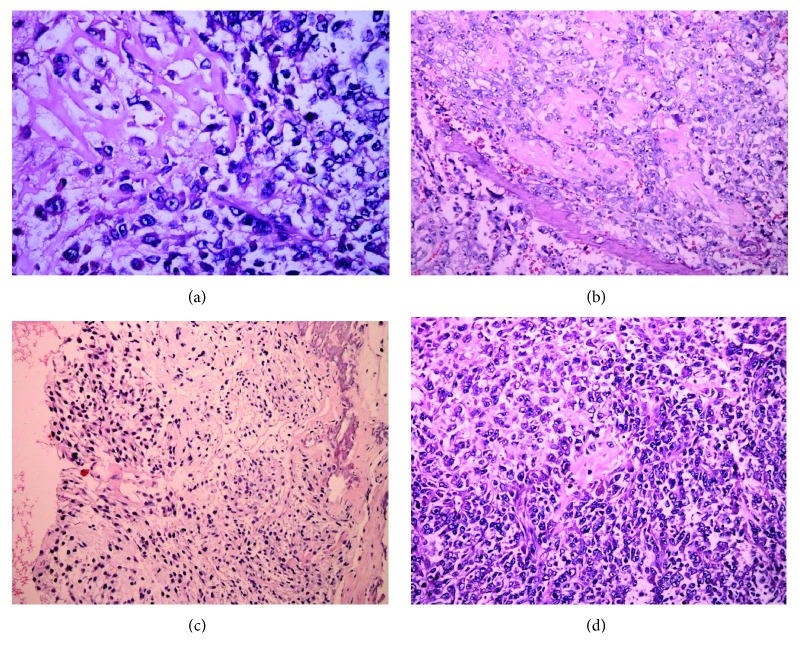
H&E staining was also performed on osteosarcoma samples at different stages. (a) The osteosarcoma consists of an atypical round to cell proliferation with osteoid deposition at G1 stage (H&E stain ×200). (b) Osteosarcoma cells shape variable with chromatic nuclei and mitosis fields at G2 stages. (c) Spindle cell neoplasm has high cellularity, abnormal mitotic characters, and atypical nuclear at the G3 stage. (d) The osteosarcoma forms a highly cancerous and high-mortality bone tumor at the G4 stage.

Figure 5 shows osteosarcoma samples with H&E staining at different stages. At the G1 stage, the osteosarcoma consisted of an atypical round to cell proliferation with osteoid deposition at the G1 stage and showed typical characters of osteosarcoma (Figure 5(a)). At the G2 stage, the shape of osteosarcoma cells was variable with chromatic nuclei and mitosis fields (Figure 5(b)). At the G3 stage, spindle cell neoplasm had high cellularity, abnormal mitotic characters, and atypical nuclei (Figure 5(c)). At the G4 stage, the osteosarcoma cells formed a highly cancerous and high-mortality bone tumor (Figure 5(d)).

### 3.4. The Plasma and Urine Levels of Naringenin

Before the therapy, the statistical difference was insignificant for urine and plasma levels of naringenin between NG and CG groups (Table 4, *P* > 0.05). In contrast, after the therapy, the statistical differences were significant for urine and plasma levels of naringenin between NG and CG groups (Table 4, *P* < 0.05). The results suggest that naringenin consumption increased urine and plasma level of the chemical.

### 3.5. Long-Term Consumption of Naringenin Controls Osteosarcoma Size

Before surgery as well as after surgery, the statistical differences between the NG and CG for clinical data (*P* > 0.05) and tumor volume were insignificant (*P* = 0.68). After an average two-year follow-up, two patients and three patients died from NG and CG, respectively. The mean tumor volumes were 106.3 ± 92.7 c.c. in the NG group as compared with 225.8 ± 141.4 c.c. in the CG group (Table 5, *P* < 0.05). The results suggest that long-term consumption of naringenin controls osteosarcoma size. On the other hand, naringenin had no impact on osteosarcoma metastasis between NG and CG groups (Table 5, *P* > 0.05).

### 3.6. Long-Term Consumption of Naringenin Reduces the Local Recurrence of Osteosarcoma

After an average two-year follow-up, the rates of local recurrence of osteosarcoma were 38.3% and 73.0% in NG and CG groups (*P* < 0.01), respectively. The results suggest that long-term consumption of naringenin reduces the local recurrence of osteosarcoma.

### 3.7. Naringenin Improves Biochemical Indexes of Osteosarcoma Patients

Table 6 shows that naringenin improved lipid profiles when compared with CG (*P* < 0.05). Naringenin increased the level of HDL-C and reduced the levels of TG, TC, LDL-C, and MDA and reduced the serum levels of TG, TC, LDL-C, and MDA after 8 months but the statistical difference was insignificant between two groups (*P* > 0.05). Lipid patterns were improved further in the NG group after 16 months, and the statistical differences were significant between the two groups (*P* < 0.05). Similarly, lipid patterns were improved considerably in NG after 24 months and the statistical differences were significant between the two groups (*P* < 0.05). All the results suggest that naringenin improves the lipid patterns in osteosarcoma patients.

As shown in Table 7, naringenin increased SOD and GSH levels when compared with the levels in controls (*P* < 0.05). In contrast, serum levels of ALT were lower in the NG group than in the CG group (*P* < 0.05). Furthermore, long-term naringenin consumption reduced the ratios of AST/ALT when compared with the CG group (*P* < 0.05). The results suggest that long-term naringenin improves antioxidant activities of osteosarcoma patients.

### 3.8. Naringenin Consumption Significantly Reduces the Levels of IL-1 Beta and IL-6

**Figure 6:**
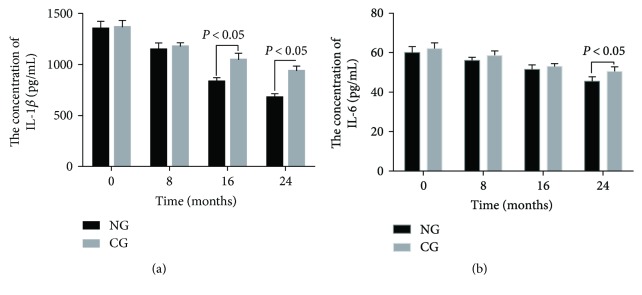
Measurement of concentrations of IL-1 beta and IL-6 by ELISA in blood samples. (a) The concentration of IL-1 beta. (b) The concentration of IL-6. All data were presented as mean values ± S.D. There are statistically significant differences if *P* < 0.05.

As Figure 6 showed, Student's *t*-test analysis showed that the statistical difference was insignificant for serum level of IL-1 beta and IL-6 between the two groups before this study (*P* > 0.05). Comparatively, the levels of IL-1 beta and IL-6 were reduced in both groups (*P* < 0.05) but the statistical difference was still insignificant for serum IL-1 beta and IL-6 between the two groups after 8 months (*P* > 0.05). The levels of IL-1 beta and IL-6 were further reduced in both groups (*P* < 0.05) and the statistical differences were significant for serum IL-1 beta between the two groups after 16 months (Figure 6(a), *P* < 0.05). Statistical differences were significant in serum IL-6 between two groups after 24 months (Figure 6(b), *P* < 0.05). The results suggest that long-term naringenin consumption significantly reduces the serum levels of IL-1 beta and IL-6.

### 3.9. Naringenin Consumption Significantly Reduces Serum ROS Levels

**Figure 7:**
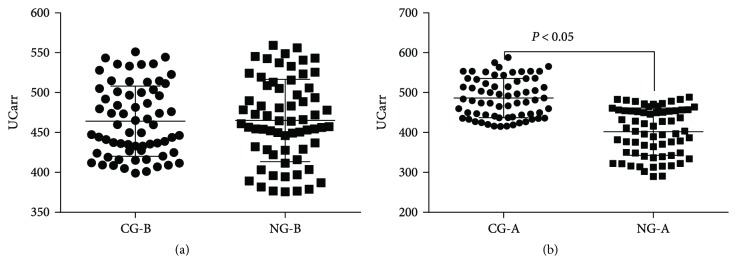
Serum ROS levels of osteosarcoma patients. CG-B, the patients before receiving placebo treatment. NG-B, the patients before receiving naringenin treatment. CG-A, the patients after receiving two-year placebo treatment. NG-B, the patients after receiving two-year naringenin treatment.

Flow cytometry analysis showed that the statistical difference was insignificant for ROS production between NG and CG groups before naringenin consumption (*P* > 0.05, Figure 7(a)). Relative ROS production was lower in the NG group than the CG group after naringenin consumption (*P* < 0.05, Figure 7(b)). Naringenin consumption remarkably reduced serum ROS levels. The results suggest that oxidative stress is still prominent in controls when compared with treated group while the oxidative stress is low after naringenin treatment.

### 3.10. Naringenin from *C. aurantium* Reduces ROS Levels of MG-63 Cells

**Figure 8:**
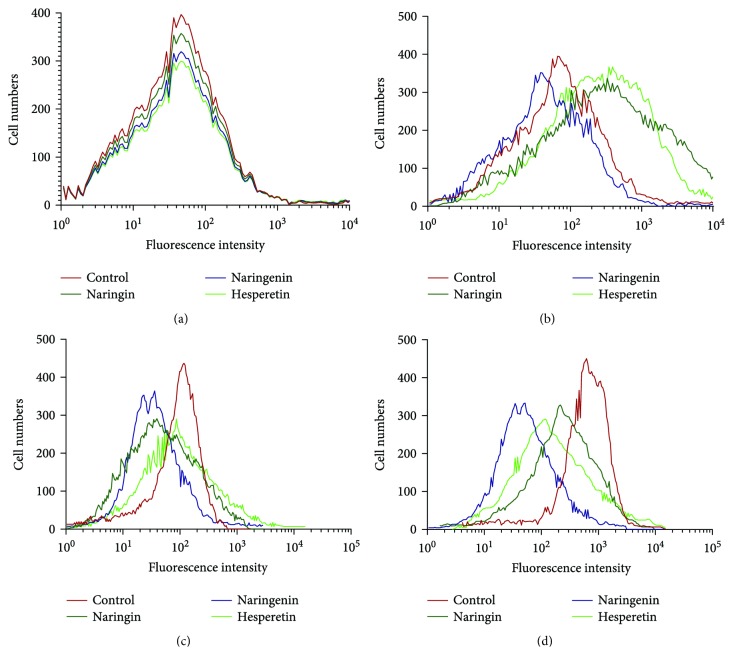
Flow cytometry analysis of relative ROS production. (a) Cell culture on 0 d. (b) Cell culture on 1 d. (c) Cell culture on 2 d. (d) Cell culture on 3 d.

Flow cytometry analysis showed that the statistical difference was insignificant for relative ROS production among the treatments of naringin, naringenin, and hesperetin when compared with the controls on 0 day (*P* > 0.05, Figure 8(a)). Relative ROS production was lower in naringenin-treated group than the control, whereas the level in the control group was lower than naringin- and hesperetin quercetin-treated groups on 1 d (*P* < 0.05, Figures 5(b) and 8). The results suggest that naringin and hesperetin treatments increase oxidative stress in the cells on 1 d (*P* < 0.05, Figure 8(b)). Relative ROS production was lower in naringenin-treated group than the control, whereas the level in controls was higher than naringin- and hesperetin-treated groups on 2 d (*P* < 0.05, Figure 8(c)). Similarly, relative ROS production was lower in naringenin-treated groups than the control, whereas the level in controls was higher than naringin- and hesperetin-treated groups on 3 d (*P* < 0.05, Figure 8(d)).

## 4. Discussion

In most countries, the treatment of osteosarcomas is delayed due to socioeconomic factors. The results lead to that osteosarcoma is difficult to be treated at advanced stages. More unfortunately, an effective therapy is still lacking for controlling osteosarcoma development. Present findings demonstrate that naringenin from *C. aurantium* has significant inhibitory effects on osteosarcoma regrowth and local recurrence following surgery in osteosarcoma. These results suggest that *C. aurantium* as functional fruit may be beneficial to prevent the risk and progression of osteosarcoma.

We have examined a single compound naringenin. The results should have the same effect with *C. aurantium* extracts regardless of the source. Furthermore, in a food or a crude extract, no other ingredients affected naringenin activity. It is well known that the case of beta-carotene in smokers turns out to be carcinogenic [43] although beta-carotene has been widely reported as a cancer suppressor [44–46]. In the case, naringenin may have reverse effects when it interacts with other components in food. Therefore, the effects of the meals of NG and CG groups, naringenin, and placebo on the growth of MG-63 cells were measured. The results indicated that crude extracts of *C. aurantium* inhibited the growth of MG-63 cells (Figure 3(d)) and had the similar results with only naringenin (Figure 3(b)). The crude extracts exerted their function via naringenin. The findings suggest that the meals of NG group cannot affect the growth of MG-63 cells (Figure 3(e)). In contrast, the meals would affect the growth of MG-63 cells when naringenin was used (Figure 3(f)). Similarly, the meals of CG group could not affect the growth of MG-63 cells (Figures 3(g) and 3(h) with placebo). In contrast, the meals would affect the growth of MG-63 cells when naringenin was used (Figure 3(i)). The results suggest it is naringenin and not the diets or other possible components affecting osteosarcoma. Therefore, *C. aurantium* may prevent the risk and progression of osteosarcoma via naringenin.

Naringin (a disaccharide derivative that is (S)-naringenin substituted by a 2-O-(*α*-L-rhamnopyranosyl)-*β*-D-glucopyranosyl moiety at position 7 via a glycosidic linkage), naringenin(a trihydroxyflavanone that is flavanone substituted by hydroxy groups at positions 5, 6, and 4′), narirutin (a disaccharide derivative that is (S)-naringenin substituted by a 6-O-(6-deoxy-*α*-L-mannopyranosyl)-*β*-D-glucopyranosyl moiety at position 7 via a glycosidic linkage), and hesperidin (a disaccharide derivative that consists of hesperetin substituted by a 6-O-(*α*-L-rhamnopyranosyl)-*β*-D-glucopyranosyl moiety at position 7 via a glycosidic linkage) are the most richly flavonoids in citrus. Because of different structures, there were great differences in pharmacokinetics of naringenin naringin and other flavonoids. For instance, when naringin is taken orally, only naringenin and predominantly its glucuronides/sulfates are circulating in blood [47]. Pure hesperidin and naringin cannot lower serum TC and LDL-C levels in moderately hypercholesterolemic patients [48]. In the present study, citrus with naringenin lowered the serum levels of TG and LDL-C (Table 6). Naringenin increased the level of HDL-C and reduced the levels of TG, TC, LDL-C, and MDA and reduced the serum levels of TG, TC, LDL-C, and MDA.

The size of osteosarcoma is an important predictive factor for osteosarcoma progression and closely associated with the developing stages of osteosarcoma (Figure 4). However, the causes of osteosarcoma are multifactorial and may be not dependent on the initial size of osteosarcoma, which was not investigated here. Naringenin from *C. aurantium* reduces the size of osteosarcoma volume when compared with the group treated with placebo, suggesting that the bioactive component can control osteosarcoma better when subsequent treatment becomes difficult. Osteosarcoma recurrence makes osteosarcoma removal very difficult although surgery is performed. Naringenin from *C. aurantium* also reduces the recurrence of residual osteosarcoma after surgery, suggesting the new drug is a potential adjuvant for osteosarcoma therapy.

Naringenin has been reported to have potential anti-inflammatory [49] and anticarcinogenic [50] applications and to attenuate lipid peroxidation [51] and gastrointestinal permeability [52]. Furthermore, naringenin has anti-inflammatory properties in obesity therapy [53]. Present findings also demonstrate that naringenin is a main compound in *C. aurantium* and has significant anti-inflammatory (Figure 6) and antioxidant (Table 7) activities for osteosarcoma progression.

ROS level plays an important role in the risk of cancer, so the level was measured in MG-63 cells. The effects of naringin, naringenin, and hesperetin on relative ROS products were also investigated. The results showed that relative ROS production was increased with the culture of MG-63 cells (Figure 7). Relative ROS production was increased in naringin- and hesperetin-treated groups after one-day culture (Figure 7(b)) while the level was always lowest in three-day culture in the naringenin-treated group (Figure 7). All the results suggest that relative ROS level is increased with the cell development while naringenin can reduce ROS level well in the cells. Thus, the similar results for naringenin controlling ROS level were obtained in patients or at a cell level. The results from cell test support the possible mechanism for naringenin function in patients.

In real life, heterogeneous populations are very common. To reduce the interference, all subjects were inquired to come from the same city (Changchun, China) with similar economic and cultural background. Naringenin did not cause remarkable side effects in the follow-up because low dose (20 mg) was used daily. Food adjustments may be a problem because it was performed according to personal situation. To control the difference, daily food consumption between NG and CG groups was maintained at similar level (Table 3). General quality of life was evaluated with the Short Form-36 (SF-36) [54]. The statistical difference was insignificant between the two groups during the follow-up (*P* > 0.05).

There are some limitations to the present work: (1) the sample size still seems small to confirm the complex situations of osteosarcoma therapy and (2) amputation is often considered for osteosarcoma patients and not considered here because the surgery will affect the study on osteosarcoma recurrence. In the case, the effects of naringenin on these patients are still unknown; (3) the extracts of naringenin from *C. aurantium* are rich with various bioflavonoids, which can be determined in the present study with a simple isolation method. More difficultly, naringenin cannot be made on a larger scale although its contents are significant in *C. aurantium* and the preparation of naringenin is still at small scale and limits its use in osteosarcoma therapy; (4) this is just a single cell line study. All the effects the authors observed may be specific to one cell line; (5) residual osteosarcoma could not be removed completely at the start of the present experiment because the surgery needs a high skill. In that case, residual osteosarcoma will affect subsequent experimental results. Therefore, much work is further needed to address these problems in the future.

## 5. Conclusions

Naringenin is a main component of *C. aurantium* and can control osteosarcoma size and osteosarcoma recurrence. Furthermore, a food or a crude extract has no synergistic, additive, and possibly antagonistic effects on naringenin activity. On the other hand, naringenin has beneficial effects on osteosarcoma patients as follows: it can improve antioxidant capacities of osteosarcoma patients by increasing the serum levels of SOD and GSH and reducing the serum levels of AST and ALT; it can improve lipid profile of osteosarcoma patients by improving the serum levels of HDL-C and reducing the serum levels of TG, TC, LDL-C, and MDA; and it can improve the anti-inflammatory capacities of osteosarcoma patients by reducing the serum levels of IL-1 beta and IL-6. More importantly, naringenin from *C. aurantium* inhibits osteosarcoma volume and recurrence in the patients who underwent surgery by reducing ROS levels. These findings suggest that naringenin from *C. aurantium* should be developed as a new drug for osteosarcoma therapy due to its antioxidant properties.

## Figures and Tables

**Table 1:** Chemotherapy for osteosarcoma patients in the present study.

Drug dose		Flowchart	
Drug	Drug, mg/m^2^/cycle	Day	Drug
(1) Neoadjuvant chemotherapy (3 cycles)		First cycle	
DDP	80	1	DDP
CTX	540	3	CTX + VCR
VCR	1	15	ADR
ADR	40	16	ADR
(2) Surgery		Second cycle	
(3) Adjuvant chemotherapy (4 cycles)		29	DDP
DDP	80	31	CTX + VCR
CTX	540	43	ADR
VCR	1	44	ADR
ADR	40	And so on	

DDP = cisplatin; CTX = cyclophosphamide; VCR = vincristine; ADR = adriamycin.

**Table 2:** Baseline characters of osteosarcoma patients.

	Naringenin group (*n* = 47)	Control group (*n* = 48)	*P* values
Age, years	23.6 ± 12.7	24.3 ± 12.1	0.21
Gender, male/female	27/20	29/19	0.77
Smoking, *n* (%)	19 (40.4)	21 (43.8)	0.74
Alcoholic intake, *n* (%)	17 (36.2)	18 (37.5)	0.89
Body mass index, kg/m^2^	25.6 ± 6.9	24.9 ± 7.2	0.14
Osteosarcoma types			
Intramedullary osteosarcoma	39 (83.0)	38 (79.2)	0.64
Juxtacortical osteosarcoma	7 (14.9)	8 (16.7)	0.81
Extraskeletal osteosarcoma	1 (21.3)	1 (41.7)	1.00
Osteosarcoma grades			
G1, *n* (%)	8 (17)	7 (14.6)	0.75
G2, *n* (%)	9 (19.1)	10 (20.8)	0.84
G3, *n* (%)	22 (46.8)	24 (50)	0.76
G4, *n* (%)	8 (17)	7 (14.6)	0.75
Osteosarcoma volume			
Before surgery	248.7 ± 124.3 c.c.	260.5 ± 113.7 c.c.	0.13
After surgery	3.9 ± 3.9 c.c.	3.5 ± 3.5 c.c.	0.24
Chemotherapy, *n* (%)	35 (74.5)	33 (68.8)	0.77
Resection length, cm	13.4 ± 5.9	12.7 ± 6.2	0.25
Stem diameter, cm	11.7 ± 3.6	10.5 ± 4.3	0.16
Site			
Cancer treatment types			
Osteosarcoma surgery	47 (100)	48 (100)	1.00
Chemotherapy	47 (100)	48 (100)	1.00
Radiotherapy	5 (10.6)	6 (12.5)	0.78
Rehabilitation and supportive care	47 (100)	48 (100)	1.00
Femur, *n* (%)	31 (66)	30 (62.5)	0.73
Tibia, *n* (%)	12 (25.5)	11 (22.9)	0.77
Others, *n* (%)	4 (8.5)	7 (14.6)	0.36
Histologic type			
Osteoblastic, *n* (%)	20 (42.6)	18 (37.5)	0.62
Chondroblastic, *n* (%)	14 (29.8)	17 (35.4)	0.56
Fibroblastic, *n* (%)	9 (19.1)	8 (16.7)	0.75
Others, *n* (%)	4 (8.5)	5 (10.4)	0.97
Differentiation status			
High, *n* (%)	31 (66)	33 (68.8)	0.71
Low, *n* (%)	16 (34)	15 (31.3)	0.77
Pulmonary metastasis, *n* (%)	4 (8.5)	5 (10.4)	0.97

**Table 3:** The comparison for daily food consumption between NG and CG groups (g/day).

Food items	NG	CG	*t* value	*P* values
Fruits				
Citrus	15.9 ± 8.6	13.4 ± 8.1	0.35	0.48
Apple, plum, pear, peach	28.2 ± 13.5	23.3 ± 14.7	0.96	0.21
Banana	15.9 ± 5.8	14.7 ± 6.1	0.17	0.92
Grape	12.1 ± 5.4	11.9 ± 5.0	0.72	0.15
Litchi	9.5 ± 4.3	8.8 ± 4.6	0.32	0.65
Mango, persimmon	3.6 ± 1.6	3.8 ± 1.2	0.00	0.50
Papaya	10.3 ± 2.3	9.6 ± 1.8	0.52	0.31
Watermelon	19.1 ± 14.5	22.7 ± 12.8	0.19	0.12
Vegetables				
Soybean	12.3 ± 6.5	13.6 ± 4.7	0.46	0.29
Kale, broccoli	21.5 ± 17.3	22.9 ± 20.1	0.26	0.47
Lettuces	19.1 ± 10.7	17.1 ± 9.8	0.12	0.45
Amaranth, spinach	26.0 ± 17.5	28.5 ± 16.2	0.29	0.18
Chinese cabbage	35.6 ± 14.2	33.8 ± 12.9	0.67	0.13
Onion and garlic	15.2 ± 7.4	16.3 ± 7.5	0.29	0.63
Eggplant, radish	30.6 ± 20.4	28.1 ± 18.7	0.08	0.55
Tomato	33.9 ± 20.5	36.8 ± 23.5	0.64	0.07
Pepper	11.7 ± 6.9	10.9 ± 7.2	0.21	0.26
Lotus root, taro	28.2 ± 6.5	27.4 ± 6.9	0.27	0.15
Tofu	16.8 ± 11.2	17.4 ± 10.9	0.18	0.47
Bean curd	3.8 ± 4.2	4.1 ± 3.5	0.05	0.50
Mushroom	12.4 ± 6.5	11.4 ± 5.4	0.48	0.32
Seed and nuts				
Sunflower	5.7 ± 3.6	6.0 ± 2.4	0.31	0.66
Peanut	7.8 ± 3.2	7.1 ± 4.1	0.22	0.75
Cashew	3.2 ± 1.4	3.6 ± 2.0	0.16	0.78
Protein food				
Beef	16.4 ± 8.7	17.2 ± 6.1	0.92	0.18
Lamb	8.5 ± 4.3	8.1 ± 4.9	0.65	0.37
Pork	26.4 ± 12.8	28.6 ± 13.1	0.87	0.24
Fish	18.2 ± 11.6	17.3 ± 9.9	0.54	0.42
Eggs	21.2 ± 12.3	19.7 ± 14.0	0.73	0.39
Poultry	16.5 ± 8.4	14.9 ± 9.2	1.43	0.07
Carbohydrate				
Barley	17.6 ± 11.5	15.3 ± 10.4	1.28	0.15
Rice	28.4 ± 17.9	30.2 ± 16.7	0.94	0.20
Corn	25.6 ± 15.4	26.8 ± 14.3	0.11	0.86
Oats	10.2 ± 6.5	11.4 ± 5.1	0.58	0.33
Sugar	5.5 ± 4.1	5.0 ± 3.8	0.20	0.76
Taro	12.7 ± 5.2	11.6 ± 6.0	0.65	0.26
Potato	18.3 ± 6.7	19.4 ± 7.2	1.31	0.11
Wheat	30.4 ± 14.2	27.9 ± 15.6	0.69	0.25
Animal fats	22.5 ± 13.8	20.6 ± 11.9	0.28	0.39
Vegetable oils	10.7 ± 4.2	9.6 ± 5.1	0.31	0.26
Beverage (mL/d)				
Milk	100.0 ± 50.0	100.0 ± 50.0	0	0.50
Coffee	64.0 ± 25.0	58.0 ± 22.0	0.68	0.15
Tea	55.0 ± 35.0	60.0 ± 34.0	0.34	0.64
Juice	120.0 ± 50.0	110.0 ± 50.0	0.13	0.76
Soft drinks	100.0 ± 50.0	100.0 ± 50.0	0	0.50
Basic diet macronutrient composition				
Energy (kcal/d)	1423.3 ± 406.5	1570.6 ± 532.1	0.54	0.30
Total carbohydrate (g/d)	201.4 ± 76.8	195.6 ± 92.4	0.24	0.69
Protein (g/d)	71.2 ± 26.2	68.4 ± 27.5	0.34	0.23
Dietary fiber (g/d)	14.7 ± 5.2	13.8 ± 5.6	0.18	0.71
Vitamin E (mg/d)	12.6 ± 5.5	11.2 ± 4.3	0.44	0.19
Vitamin C (mg/d)	139.6 ± 71.8	130.4 ± 65.7	1.12	0.15

Note: *t*-test calculator for two independent means. NG: naringenin group; CG: control group with placebo.

**Table 4:** The plasma and urine levels of naringenin (ng/mL).

		NG	CG	*P* value
Plasma	Before therapy	3.47 ± 1.58	3.69 ± 1.74	0.23
	After therapy	82.16 ± 18.37	3.23 ± 1.62	0.01^∗^

Urine	Before therapy	1.01 ± 0.05	1.06 ± 0.07	0.12
	After therapy	22.31 ± 9.45	1.17 ± 0.09	0.01^∗^

Note: *t*-test calculator for two independent means. ^∗^*P* < 0.05 via CG.

**Table 5:** Osteosarcoma volume and metastasis after average two-year follow-up.

	Naringenin group (*n* = 45)	Control group (*n* = 45)	*P* values
Osteosarcoma volume	106.3 ± 92.7 c.c.	225.8 ± 141.4 c.c.	0.01^∗^
Pulmonary metastasis, *n* (%)	7 (15.6)	9 (20.0)	0.58
Other bone metastasis, *n* (%)	5 (11.1)	6 (13.3)	0.75

Note: 2 died in naringenin group and 3 died in control group after a two-year follow-up. ^∗^*P* < 0.05 via a control group.

**Table 6:** Comparison of lipid pattern in osteosarcoma patients before and after therapy.

		TG (mmol/L)	TC (mmol/L)	HDL-C (mmol/L)	LDL-C (mmol/L)	MDA (mmol/L)
Before	NG	2.8 ± 1.2	5.7 ± 1.2	1.3 ± 0.3	3.8 ± 1.0	1.7 ± 0.3
	CG	2.7 ± 1.1	5.6 ± 1.2	1.2 ± 0.2	4.0 ± 1.1	1.6 ± 0.2
	*P* value	0.73	0.86	0.64	0.76	0.89

8-month	NG	2.6 ± 1.3	5.5 ± 1.1	1.4 ± 0.2	3.5 ± 1.1	1.5 ± 0.2
	CG	2.7 ± 1.3	5.7 ± 1.1	1.3 ± 0.2	3.9 ± 1.1	1.7 ± 0.3
	*P* value	0.34	0.22	0.18	0.26	0.27

16-month	NG	2.2 ± 1.3	5.1 ± 1.1	1.6 ± 0.2	3.2 ± 1.1	1.3 ± 0.2
	CG	2.8 ± 1.3	5.8 ± 1.1	1.3 ± 0.2	4.0 ± 1.2	1.8 ± 0.3
	*P* value	0.02^∗^	0.04^∗^	0.04^∗^	0.03^∗^	0.02^∗^

24-month	NG	1.8 ± 1.1	4.6 ± 1.4	1.6 ± 0.3	3.0 ± 1.1	0.9 ± 0.1
	CG	2.6 ± 1.3	5.5 ± 1.1	1.2 ± 0.2	3.9 ± 1.2	1.6 ± 0.3
	*P* value	0.01^∗^	0.02^∗^	0.01^∗^	0.01^∗^	0.01^∗^

Note: *t*-test calculator for two independent means. ^∗^*P* < 0.05 via CG.

**Table 7:** Biochemical parameters of enzyme activities.

		SOD (U/mL)	GSH (ng/mL)	ALT (U/mL)	AST (U/mL)	AST/ALT
Before	NG	25.34 ± 3.17	26.89 ± 4.19	46.88 ± 12.24	42.34 ± 15.26	0.91 ± 0.16
	CG	22.78 ± 3.02	25.65 ± 4.04	48.31 ± 11.38	44.18 ± 12.39	0.92 ± 0.18
	*P* value	0.23	0.56	0.14	0.40	0.56

8-month	NG	26.38 ± 2.90	27.45 ± 4.27	45.84 ± 15.23	36.35 ± 11.43	0.81 ± 0.13
	CG	25.31 ± 3.24	29.36 ± 4.78	43.22 ± 16.51	38.48 ± 10.55	0.88 ± 0.15
	*P* value	0.64	0.12	0.08	0.17	0.11

16-month	NG	25.44 ± 2.37	26.99 ± 4.91	43.24 ± 16.98	32.48 ± 9.36	0.74 ± 0.12
	CG	30.32 ± 3.24	35.38 ± 4.56	38.46 ± 17.25	40.23 ± 11.22	1.01 ± 0.17
	*P* value	0.02^∗^	0.04^∗^	0.04^∗^	0.01^∗^	0.01^∗^

24-month	NG	29.49 ± 2.93	32.11 ± 5.27	40.35 ± 18.29	28.40 ± 10.44	0.69 ± 0.12
	CG	46.39 ± 3.89	39.71 ± 4.56	30.28 ± 19.16	41.95 ± 18.32	1.42 ± 0.21
	*P* value	0.01^∗^	0.02^∗^	0.01^∗^	0.01^∗^	0.01^∗^

Note: ^∗^*P* < 0.05 versus CG.
